# Population Size Influences Amphibian Detection Probability: Implications for Biodiversity Monitoring Programs

**DOI:** 10.1371/journal.pone.0028244

**Published:** 2011-12-02

**Authors:** Lorenzo G. Tanadini, Benedikt R. Schmidt

**Affiliations:** 1 Institute of Evolutionary Biology and Environmental Studies, University of Zurich, Zurich, Switzerland; 2 KARCH, Neuchâtel, Switzerland; Smithsonian's National Zoological Park, United States of America

## Abstract

Monitoring is an integral part of species conservation. Monitoring programs must take imperfect detection of species into account in order to be reliable. Theory suggests that detection probability may be determined by population size but this relationship has not yet been assessed empirically. Population size is particularly important because it may induce heterogeneity in detection probability and thereby cause bias in estimates of biodiversity. We used a site occupancy model to analyse data from a volunteer-based amphibian monitoring program to assess how well different variables explain variation in detection probability. An index to population size best explained detection probabilities for four out of six species (to avoid circular reasoning, we used the count of individuals at a previous site visit as an index to current population size). The relationship between the population index and detection probability was positive. Commonly used weather variables best explained detection probabilities for two out of six species. Estimates of site occupancy probabilities differed depending on whether the population index was or was not used to model detection probability. The relationship between the population index and detectability has implications for the design of monitoring and species conservation. Most importantly, because many small populations are likely to be overlooked, monitoring programs should be designed in such a way that small populations are not overlooked. The results also imply that methods cannot be standardized in such a way that detection probabilities are constant. As we have shown here, one can easily account for variation in population size in the analysis of data from long-term monitoring programs by using counts of individuals from surveys at the same site in previous years. Accounting for variation in population size is important because it can affect the results of long-term monitoring programs and ultimately the conservation of imperiled species.

## Introduction

In order to assess distribution and abundance of species, conservationists have initiated long-term monitoring programs. Monitoring programs are an important element in the toolbox of conservationists because they are often used to determine whether conservation measures were successful, to evaluate the efficiency of management policy and ultimately to decide where conservation funds should be allocated [1]–[4].

There are various sources of error that can cause bias in estimates of distribution and abundance [5]–[6]. One source of error is imperfect detection on individuals, populations, or species [1], [7]–[8]. Imperfect detection means that individuals, populations or species are not always found even when they are present at a site. Imperfect detection will therefore cause negative bias in estimates of abundance, distribution or species richness unless imperfect detection is accounted for [8]. Furthermore, if imperfect detection varies spatially or temporally, then spatial or temporal patterns in abundance, distribution and biodiversity can appear, even though in reality they are mere sampling artefacts [1], [8]–[9].

Therefore, state-of-the-art monitoring programs should aim at quantifying imperfect detection. That is, they estimate detection probabilities in order to avoid biases in biodiversity estimates [1], [6], [10]. Many analyses have revealed determinants of detection probabilities [11]–[18]. In addition to removing bias in biodiversity estimates, modelling and understanding determinants of detection probability has several practical advantages. If one knows when detection probabilities are highest, then field crews can be instructed when to do field work. Moreover, if all field crews conduct field work under similar conditions (e.g., weather), then this may lead to standardization of methods. Standardization is clearly an asset in monitoring programs because heterogeneity of results is reduced. This increases data quality because detection probabilities may be relatively constant [8], [19].

Most analyses that attempted to identify determinants of detection probabilities looked at factors such as weather, phenology, observer experience and survey duration. While these factors clearly may affect detection probabilities, these studies largely overlooked a potentially important source of variation in detection probabilities: population size [20]. If population size determines detection probabilities of a population, then it should be included in statistical analysis of the data because it induces heterogeneity and therefore bias in parameter estimates [21]. In addition, population-size dependent detection probabilities have clear conservation implications since small populations are more likely to be overlooked. Royle and Nichols [22] and Peterson and Bayley [23] explained how population size affects detection probability of a population. They showed that the probability of detecting a population on a sampling unit, *p*, can be written as
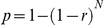
where *r* is the detection probability of an individual and *N* is abundance [22], [23]. This simple equation establishes a clear link between abundance and detection probability of a population. To date it is unknown, however, whether the effect of abundance on detectability is stronger than the effect of, say, weather conditions or habitat characteristics on abundance. For example, if weather conditions are unfavourable then few animals may be active and thus detectable. Thus, environmental conditions may cause variation in apparent abundance (the number of individuals that are active and can be detected) that may be more important than variation in true abundance.

Our goal was to compare various likely determinants of detection probability, namely an index to population size, weather conditions and habitat characteristics. To do so, we analyse data from a volunteer-based amphibian monitoring program of the Swiss canton Aargau [24], [25]. Data from an amphibian monitoring program are particularly suitable because some species –frogs and toads– vocalize whereas others are mute (the newts). We expected that for vocalizing species the population index may not matter much because a single calling male would already indicate the presence of the species. Newts, in contrast, must be actively searched such that abundance is expected to be most important.

## Materials and Methods

### Study area and field methods

The volunteer-based amphibian monitoring program of the Swiss Canton Aargau has been run since 1999. It has the goal to survey status and population trend of summer breeding endangered amphibian species [24], [25]. The Canton is subdivided into ten core areas, each one comprising ∼30 breeding sites and representing a spatial hotspot of amphibian diversity. Every year, two to three core areas out of ten are selected randomly. Therefore, not all core areas are monitored every year. Within each selected core area, all amphibian breeding sites are visited three times during that year. The first two visits are done at night during April and May, the third visit during the day in the months of June or July. A single, trained volunteer, who is usually responsible for five to ten breeding sites every year, does all three visits to a given site. Volunteers record anurans by walking along the water's edge and noting visual encounters and calls. Newts are actively searched with nets in addition to visual encounters. The survey is done accordingly to a standardised protocol, which stipulates precise time rules for the visit of each site according to its size. Volunteers report counts of all life history stages (eggs, larvae, juveniles, adults) of all amphibian species encountered and the date and time when they undertook the site visits. Volunteers also have to describe some amphibian breeding site (i.e., pond) characteristics regarding vegetation state and site structure during the third (daytime) survey.

### Data

This study is based on the data gathered within the frame of the monitoring program in the years 1999–2006. Out of these years, we selected only the most recent and the second most recent survey available for each amphibian breeding site. The most recent survey available is generally from the years 2004–2006, and the second-most recent survey available for the same amphibian breeding site was on average conducted two years earlier. For our analysis, we used all amphibian breeding sites for which data on amphibians and site characteristics was complete (i.e., no missing values; *n* = 165).

We selected six amphibian species that allowed interesting comparisons among species: loud calls vs. quiet calls and newts vs. anurans. The species were: the midwife toad *Alytes obstetricans*, the yellow-bellied toad *Bombina variegata*, the natterjack toad *Bufo calamita*, frogs of the water frog *Pelophylax esculentus*-complex, and the two newts alpine newt *Mesotriton alpestris*, and crested newt *Triturus cristatus* (nomenclature follows [26]). Every species was analysed individually. Although volunteers report counts of all life history stages for all species, this analysis is based on adult counts only. Adult counts usually underestimate true abundance but there is a positive correlation between true abundance and the counts; the counts may thus serve as a useful index to amphibian population size [19].

Weather data was provided by the Swiss Meteorological Service MeteoSchweiz (www.meteoschweiz.admin.ch). We used data from the weather station Buchs-Aarau, which is located in the center of the study area. Data on amphibian breeding site characteristics were collected during the amphibian surveys.

### Data analysis

We evaluated the explanatory power of different covariates on detection probability by adding covariates to the mark-recapture-like site occupancy models developed by MacKenzie et al. [27] and Tyre et al. [11]. This approach assigns a “detection history” to each site, which can be read as a vector of ones and zeroes of length equal to the number of surveys at a specific site. The “1” indicates that at least one individual of the target species was observed, and the “0” that no individuals were observed. The model assumes that the system is closed during the surveys, i.e. no populations go extinct or empty patches are colonized. The detection history for an amphibian breeding site visited three times could be 1 1 0 (i.e., the species was detected during the first two surveys but not during the third), and the corresponding probability for the detection history would be defined as *Ψ p*_1_ *p*_2_ (1−*p*_3_), where *Ψ* is the probability of site occupancy and *p*_i_ is the detection probability for visit *i*. The product of all probabilities forms a model likelihood for the observed data set. Estimates of the probability of site occupancy and detection probability can be obtained by maximizing the likelihood function. If both occupancy and detection probabilities are constant, the model likelihood can be written as [27]:
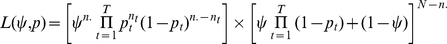
where *ψ_i_* is the probability that a species is present, *p_it_* is the probability will be detected at site *i* at time *t*, given presence, *N* is the total number of surveyed sites, *T* the number of distinct sampling occasions, *n_t_* the number of sites where the species was detected at time *t* and *n*. the total number of sites at which then species was detected at least once [27]. We did not fit the Royle-Nichols [22] model (which uses the equation mentioned in the Introduction) to the data because detection of species was often based on different life history stages. One cannot assume that the relationship described in the equation in the Introduction holds if detection/non-detection data is based on different life history stages.

The data analysis was carried out with the statistical software PRESENCE 2.0, which allows the estimation of the detection probability, and the site occupancy in relation to different covariates [27]. We used the small sample Akaike information criterion (AICc), ΔAICc and Akaike weights [28] to rank candidate models. We considered models as well supported by the data if their Akaike weight was greater than 0.05. Sample size was the number of amphibian breeding sites included in the analysis (*n* = 165).

### Candidate models

For each species, we fitted a small number of candidate models to the data. Models had a common intercept for all three surveys, a single covariate per model for the two night-time surveys, and also a single, but different constant term for the third daytime survey. This yielded logistic regression models of the form:
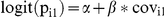


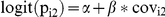


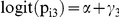
where p_it_ is the detection probability and cov_it_ is an explanatory covariate at site *i* during survey *t*. α, β and γ are the parameters (intercept and slopes) of the logistic regression. Site occupancy, which was not the focus of this study, was modeled as a constant term.

To determine which covariates explained detection probability best, we used covariates that explained the data well in previous, similar analyses (e.g., [14], [16], [18], [25]), but also some novel ones. Previously used covariates include pond characteristics (water surface, reed cover, floating aquatic vegetation, submerged vegetation, accessibility of the pond), phenology and weather covariates (wind speed, air temperature, rain). Novel covariates included the index to past population size and soil temperature. The set of candidate models was adapted to the natural history of the species (e.g. [29]). For the two newts, we did not include “wind” in the set of candidate models because they are rarely exposed to wind during their aquatic phase. For the three toads, we did not consider the covariates describing pond vegetation since they either call on land or prefer early successional ponds with little vegetation.

To determine whether population size affects detection probability, we used the count of adult individuals recorded during the most recent survey (one to six years ago) at the same amphibian breeding site [number of adult individuals] as an index to past population size (PASTPOP). Covariates that depend on species detection must not be used to model detection. Hence, the current count cannot be used (see [27]). However, past population index can be used. This is analogous to the use of previous captures as a covariate for detection probability in mark-recapture models [30], [31]. We used PASTPOP alone and also combined with the time elapsed since the most recent survey [years] (TIMESINCE). We modelled seasonality using day-of-the-year [January 1^st^ = 1] (VISIT). To allow for a peak of detection probability during the season, we also included a quadratic day-of-the-year term (VISITSQ).

Several pond characteristics were included in the analysis: pond water surface [m^2^] (WSURFACE), the percentage of the pond shore covered by reeds (mostly *Typha* sp.) [%] (REED), the percentage of the water surface covered with floating aquatic vegetation (e.g. *Potamogeton* sp. and *Nuphar* sp.) [%] (FLOATINGP), the percentage of the pond covered with submersed vegetation (e.g. *Potamogeton* sp., *Myriophyllum* sp., *Hippuris* sp. and *Elodea* sp.) and underwater plants [%] (UNDERWP), as well as a binary covariate describing the accessibility of the pond to volunteers (RESTRICTED).

We modelled the effects of several weather covariates on detection probability: wind speed [km/h] (WIND; not used for the newts), soil temperature measured at a soil depth of 5 cm [°C] (SOILT), air temperature measured at 2 meters above the ground [°C] (AIRT), as well as the amount of rain on the survey day [mm] (RAIN). Continuous covariates were standardised before analysis to enhance convergence.

## Results

Table 1 shows a summary of the model selection results: Only models with an Akaike weight greater than 0.1 are shown. Table S1 shows the full model selection results.

**Table 1:** Summary of model selection results for each species.

Model	ΔAICc^a^	Akaike weight^b^	K^c^	2log-likelihood
*A. obstetricans*				
psi(.), p(SOILT)	0.00	0.347	4	279.78
psi(.), p(AIRT)	0.18	0.317	4	279.95
psi(.), p(PASTPOP)	2.35	0.107	4	282.12
*B. variegata*				
psi(.), p(WIND)	0.00	0.518	4	289.22
psi(.), p(AIRT)	0.57	0.389	4	298.80
*P. esculentus*				
psi(.), p(PASTPOP, TIMESINCE)	0.00	0.579	5	424.85
psi(.), p(PASTPOP)	0.64	0.420	4	427.49
*B. calamita*				
psi(.), p(PASTPOP, TIMESINCE)	0.00	0.255	5	167.59
psi(.), p(PASTPOP)	1.00	0.154	4	170.58
psi(.), p(SOILT)	1.48	0.121	4	171.07
psi(.), p(AIRT)	1.62	0.113	4	171.20
*M. alpestris*				
psi(.), p(PASTPOP)	0.00	0.236	4	513.53
psi(.), p(VISIT, VISITSQ)	1.03	0.141	5	512.55
psi(.), p(SOILT)	1.06	0.139	4	514.59
psi(.), p(PASTPOP, TIMESINCE)	1.15	0.133	5	512.67
psi(.), p(RAIN)	1.66	0.103	4	515.19
*T. cristatus*				
psi(.), p(PASTPOP, TIMESINCE)	0.00	0.738	5	112.12
psi(.), p(PASTPOP)	3.17	0.151	4	117.29

a ΔAICc is the difference between the AICc of the best model and the focal model.

b K is the number of parameters included in the model.

c The sum of all Akaike weights in a set of candidate models is 1. The higher the weight, the better the model is supported by the data.

### *Alytes obstetricans*

**Figure 1:**
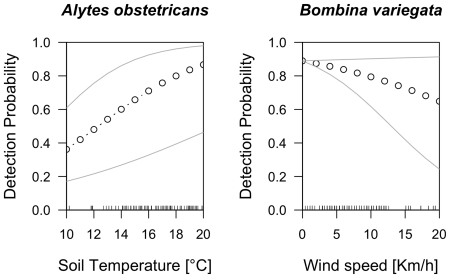
The relationship between meteorological variables and detection probabilities in two anurans. Thin gray lines are 95% confidence intervals. Small ticks inside the box indicate observed soil temperatures and wind speeds, respectively.

Two models including weather covariates had Akaike weights ∼0.3 and two models past population index and phenology, respectively, had Akaike weights ∼0.1 (Table 1). Together, they accounted for almost 85% of the Akaike weight. The model best supported by the data included the variable describing soil temperature. For this model, soil temperature had a positive effect on detection probability (slope estimate ± SE on the logit scale: β = 1.14±0.47) (Fig. 1). The estimate of detection probability during the daytime site visit (third site visit) was γ = 0.27±0.07 (estimate ± SE on the normal scale). The model including only past population index received weak support from the data. In this model, past populations index had a positive impact on detection probability (slope estimate ± SE on the logit scale: β = 0.68±0.50). For the best model including past population index, the estimate of the site occupancy probability was Ψ = 0.26±0.06 (estimate ± SE). For the best model without past population index, the estimate of the site occupancy probability was Ψ = 0.22±0.03 (estimate ± SE).

### *Bombina variegata*

Two models including weather covariates best explained the data (Table 1). Together, they account for almost 90% of the Akaike weight. The model including wind speed was best supported by the data (Akaike weight = 0.518; Table 1). For this model, wind speed had a negative impact on the detection probability (slope estimate ± SE on the logit scale: β = −0.39±0.35) (Fig. 1). The estimate of detection probability during the daytime site visit (third site visit) was γ = 0.43±0.08 (slope estimate ± SE on the normal scale). Models accounting for past population index were not supported by the data. For the best model including past population index, the estimate of the site occupancy probability was Ψ = 0.25±0.03 (slope estimate ± SE). For the best model without past population index, the estimate of the site occupancy probability was Ψ = 0.24±0.03 (slope estimate ± SE).

### *Pelophylax esculentus*-complex

**Figure 2:**
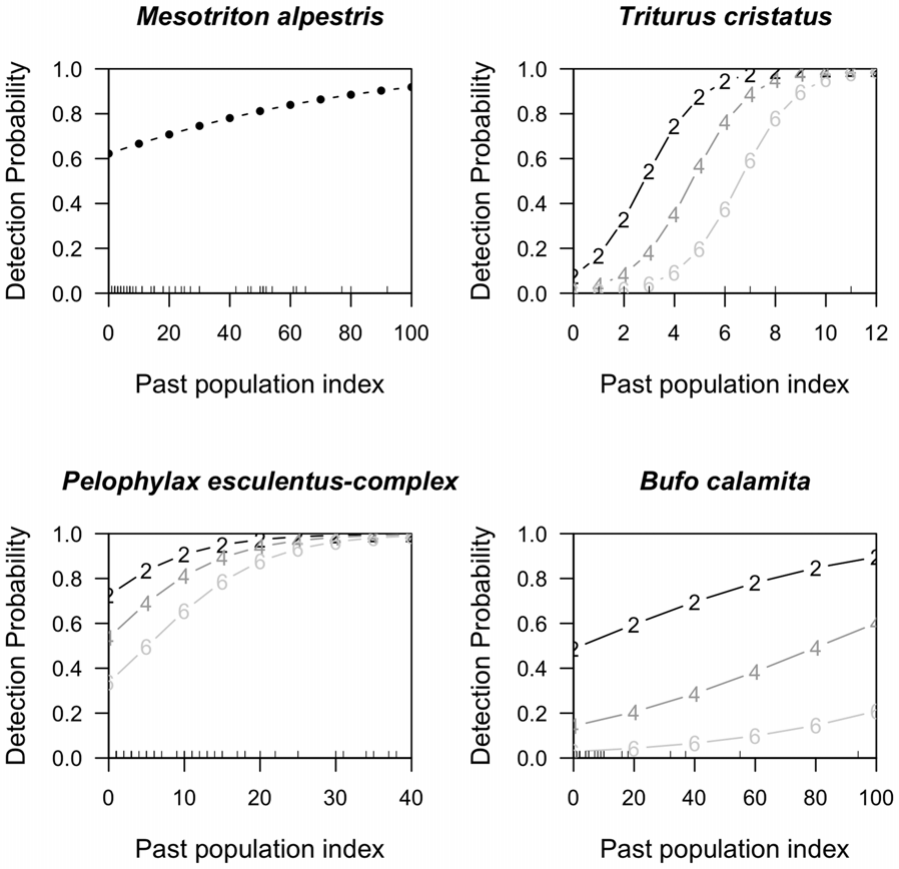
The relationship between past population index, time since last survey (two, four and six years ago) and detection probabilities in two anurans and two newts. Small ticks inside the box indicate observed population sizes.

The model including past population index (i.e., PASTPOP) and time elapsed since the last survey was best supported by the data, i.e. had the highest Akaike weight (Table 1). The second best model included only past population index. These two models accounted for almost 100% of the Akaike weight. In the best model, past population index had a positive effect on detection probability (slope estimate ± SE on the logit scale: β = 4.73±1.72), while time elapsed had a negative effect (slope estimate ± SE on the logit scale: β = −0.41±0.25) (Fig. 2). The estimate of detection probability during the daytime site visit (third site visit) was γ = 0.78±0.05 (estimate ± SE on the normal scale). For the best model including past population index, the estimate of the site occupancy probability was Ψ = 0.52±0.04 (estimate ± SE). For the best model without past population index, the estimate of the site occupancy probability was Ψ = 0.51±0.04 (estimate ± SE).

### *Bufo calamita*

Nine candidate models explained detection probability reasonably well, i.e., had Akaike weights greater than 0.05 (Table 1). The model including past population index and time elapsed since the last survey was best supported by the data. The second best model included only past population index. For the best model, past population index had a positive impact on detection probability (slope estimate ± SE on the logit scale: β = 0.27±0.26), while time elapsed had a negative effect (slope estimate ± SE on the logit scale: β = −0.87±0.50) (Fig. 2). The estimate of detection probability during the daytime site visit (third site visit) was γ = 0.20±0.09 (estimate ± SE on the normal scale). For the best model including past population index, the estimate of the site occupancy probability was Ψ = 0.15±0.04 (estimate ± SE). For the best model without past population index, the estimate of the site occupancy probability was Ψ = 0.12±0.03 (estimate ± SE).

### *Mesotriton alpestris*

Seven candidate models explained detection probability reasonably well, i.e., had Akaike weights greater than 0.05 (Table 1). They accounted for approximately 90% of the Akaike weight. The model including only past population index was best supported by the data. In this model, past population index had a positive effect on detection probability (the slope estimate ± SE on the logit scale: β = 0.40±0.21) (Fig. 2). The estimate of detection probability during the daytime site visit (third site visit) was γ = 0.16±0.04 (estimate ± SE on the normal scale). For the best model including past population index, the estimate of the site occupancy probability was Ψ = 0.66±0.05 (estimate ± SE). For the best model without past population index, the estimate of the site occupancy probability was Ψ = 0.64±0.04 (estimate ± SE).

### *Triturus cristatus*

Two models best explained the data (Table 1). Together, they account for almost 90% of the Akaike weight. Both models included past population index. The model including only past population index and time elapsed since the last survey was by far the best supported by the data. In this model, past population index had a positive effect on detection probability (slope estimate ± SE on the logit scale: β = 1.25±0.42), while time elapsed had a negative effect (slope estimate ± SE on the logit scale: β = −0.84±0.42) (Fig. 2). The estimate of detection probability during the daytime site visit (third site visit) was γ = 0.01±0.02 (estimate ± SE on the normal scale). For the best model including past population index, the estimate of the site occupancy probability was Ψ = 0.39±0.21 (estimate ± SE). For the best model without past population index, the estimate of the site occupancy probability was Ψ = 0.12±0.04 (estimate ± SE).

## Discussion

Imperfect detection is a phenomenon that all studies of distribution and abundance have to deal with [5]–[8]. An in-depth knowledge of the factors that determine detectability helps to improve parameter estimation, as well as the design of field studies and monitoring programs and ultimately leads to better species conservation. As predicted by theory [22], [23], the results of our analyses clearly show that, for a majority of species, past population index influenced detectability of amphibians in a volunteer-based monitoring program. As expected [22], [23], the effect of past population index on detection probability was positive for all species. In contrast, neither phenology, habitat nor weather conditions played an important role for most species. We believe that our results are general because we analysed data recorded by many volunteers at many amphibian breeding sites across a large area and across multiple years for amphibians with different life history characteristics. Specifically (see Table 1), past population index influenced detection probability for the species with loud calls (*P. esculentus* and *B. calamita*) but had no effect for the species with quiet calls (*A. obstetricans*, *B. variegata*). For the newt species (*M. alpestris*, *T. cristatus*), past population index influenced detection probability. We expected that past population index would matter primarily for newts that must be actively searched. For anurans, we did not expect a strong effect because even a single calling male is easy to hear and detect. Unexpectedly, our results clearly suggest that past population index matters for both visual encounter surveys and acoustic surveys. Our estimates of the effect of past population index likely underestimate the effect of (current) population size on detection probability. This is because past population size is only an index of current population size that probably underestimates true population size [19]. Nevertheless, counts and true abundance are usually positively correlated [19]. This justifies their use as an index to population size and as a covariate in our models since the goal is simply to have a covariate that describes among-site variation in abundance and adjusts detection probability in the models accordingly.

For three species, the best model included both past population index and time since last survey. The influence of population index on detection probability became smaller as the number of years between successive surveys at the same breeding site increased (Fig. 2). The importance of latter variable is straightforward to explain. Amphibian populations are known to fluctuate widely [32]–[34]. Consequently, the more time has elapsed between surveys, the less likely it is that past and present population size are highly correlated [35]. An effect of past population size is therefore likely disappear with time. We did not include models with combinations of past population index and environmental covariates in the set of candidate models. This does not affect our conclusion that past population index is an important determinant of detection probability. If such models were better than the our candidate models, they would only strengthen the case for the importance of past population index.

For two species, weather variables better explained the detection/non-detection data than past population index. In some previous studies weather variables were found to explain detection probabilities well (e.g., [14], [27], [36]). Since volunteers are told to do field work only when weather conditions are suitable for detecting amphibians (i.e. warm and moist nights), field work was done over a limited range of weather conditions. Given such a small range of weather conditions, it is unlikely that weather conditions have a strong effect on detection probability in our study. It is much more likely that they determine how many amphibians are active. For example, during warm and moist nights more male amphibians may be calling [37]. This would render the population more easily detectable. The result that detection probability of *Bombina variegata* depends on wind may be an example for such a phenomenon. In our own experience in the field, we often found that these toads are hiding during windy nights. They are only active and calling and therefore available for detection when there is no wind. This suggests that weather would only indirectly affect detection probability through its effect on the number of active individuals. Conceptually, detection probability can be decomposed into “availability for detection” and “detection probability conditional of availability for detection” [8], [38], [39]. If our explanation for an indirect effect of weather on detectability through an effect on the number of active individuals is correct, then weather would determine whether amphibians are exposed to sampling (i.e., active or inactive) and population index would determine detection probability given that toads are active and calling.

### Spatial variation in population size and the design and analysis of monitoring programs

Based on the observed relationship between detection probability and the population index, we make some comments on the design of monitoring programs. Obviously, variation in population size should be accounted for in monitoring programs. Technically speaking, variation in abundance leads to heterogeneity in detection probabilities. Such heterogeneity will lead to bias in site occupancy estimates [21]. Although counts usually underestimate true abundance [5], there is often a positive relationship between counts and abundance [19]. Thus, counting individuals may yield data that is of great importance during the analysis of monitoring data (as in this study). However, if one counts individuals anyway, then one may also directly estimate abundance and occupancy from the repeated count data [21], [40], [41]. We suggest that this may be the best approach for taxa such as birds, butterflies or reptiles where usually all members of the population are synchronously present at the sampling site and where a single life history stage (adults, pairs or territories) is counted (e.g., [42]–[44]). For other taxa, such as amphibians, the approach may be less suitable because detection may involve many life history stages such as eggs, larvae, juveniles, adults and calling males (that are not seen). Counts of these life history stages cannot be compared. Focusing on, say, only adults would probably result in lower detection probabilities and therefore poor estimates [27]. Moreover, conditions for field work might become more stringent because breeding adult amphibians may only be present at the breeding sites for a relatively short period of time. Thus, less time is available to complete field work. Given a fixed budget, one may have to reduce the number of sites that is surveyed. This, too, would lead to poorer estimates [27]. Nevertheless, some method to account for variation in abundance should be used, either using counts of past population index as a covariate or through using mixtures for modeling detection probabilities [45]. In long-term monitoring programs, the past population index is available from site visits in earlier years. In single-season surveys, mixture models may be a useful method.

The relationship between population index and detection probability suggests that small populations are more likely to be missed than large ones. If there are three visits to a site and detection probabilities of small and large populations are 0.4 and 0.8, respectively, then the probabilities to not detect a small and large population are 0.216 and 0.008, respectively. One may therefore decide to assign unequal numbers of site visits to populations that were known to be large and small in the past. For example, one may decide to visit small populations four times and large populations twice. The resulting probabilities of not detecting the populations would then be 0.1296 and 0.04, respectively. Such an unequal-number-design would probably greatly enhance the value of a long-term monitoring program because many more small populations are detected.

The dependence of detection probability on past population index implies that it is difficult to standardize field work in long-term monitoring programs. We recommend standardization of field protocols but one should keep in mind that standardization is no panacea. In particular, it is evidently impossible to “standardise” population size across sites and across years.

### Spatial variation in population size and species conservation

What are the implications of the relationship between population size and abundance that we reported in this study for the conservation of threatened species? The relationship implies that small populations are likely to be missed during surveys and in monitoring programs. This may have two consequences. First, if small populations are undetected, then they cannot be the focus of conservation action and therefore they may be more likely to go extinct (also see [46]). Second, if a population that was known to occur at a site is no longer detected because population size is small, then conservation managers may stop species-specific management actions. As a consequence, the species may go locally extinct.

### Conclusion

We do not want to deny an effect of weather or other variables on detection probabilities. Rather, we would like to emphasize that population index appears to be a predictor of detection probability that is both theoretically and intuitively appealing. As we have shown here, one can easily account for variation in population size by using counts of individuals from surveys at the same site in previous years. Accounting for variation in population size is important because it can affect the results of long-term monitoring programs and ultimately the conservation of imperiled species.

## Supporting Information

Table S1 **Models sets and model selection results for each species.** The table lists all candidate models for all species and shows the results of the model selection process (ΔAICc, Akaike weights, number of parameters (K) and -2log-likelihood).
(PDF)

## References

[1] Pollock KH, Nichols JD, Simons TR, Farnsworth GL, Bailey LL, Sauer JR (2002). Large scale wildlife monitoring studies: statistical methods for design and analysis.. Environmentrics.

[2] Weber D, Hintermann U, Zangger A (2004). Scale and trends in species richness: considerations for monitoring biological diversity for political purposes.. Global Ecology and Biogeography.

[3] Nichols JD, Williams BK (2006). Monitoring for conservation.. Trends in Ecology & Evolution.

[4] Lindenmayer DB, Likens GE (2010). Effective ecological monitoring.

[5] Anderson DR (2001). The need to get the basics right in wildlife field studies.. Wildlife Society Bulletin.

[6] Yoccoz NG, Nichols JD, Boulinier T (2001). Monitoring biological diversity in space and time.. Trends in Ecology & Evolution.

[7] Preston FW (1979). The invisible birds.. Ecology.

[8] Kéry M, Schmidt BR (2008). Imperfect detection and its consequences for monitoring for conservation.. Community Ecology.

[9] Link WA, Sauer JR (1998). Estimating population change from count data: application to the North American Breeding Bird Survey.. Ecological Applications.

[10] Kéry M, Schmid H (2004). Monitoring programs need to take into account imperfect species detectability.. Basic and Applied Ecology.

[11] Tyre AJ, Tenhumberg B, Field SA, Niejalke D, Parris K (2003). Improving precision and reducing bias in biological surveys: estimating false-negative error rates.. Ecological Applications.

[12] Bailey LL, Simons TR, Pollock KH (2004). Estimating site occupancy and species detection probability parameters for terrestrial salamanders.. Ecological Applications.

[13] Pellet J, Schmidt BR (2005). Monitoring distributions using call surveys: estimating site occupancy, detection probabilities and inferring absence.. Biological Conservation.

[14] Weir LA, Royle JA, Nanjappa P, Jung RE (2005). Modeling anuran detection and site occupancy on North American Amphibian Monitoring Program (NAAMP) routes in Maryland.. Journal of Herpetology.

[15] Mazerolle MJ, Bailey LL, Kendall WL, Royle JA, Converse SJ (2007). Making great leaps forward: Accounting for detectability in herpetological field studies.. Journal of Herpetology.

[16] Kröpfli M, Heer P, Pellet J (2010). Cost-effectiveness of two monitoring strategies for the great crested newt (*Triturus cristatus*).. Amphibia-Reptilia.

[17] McClintock BT, Bailey LL, Pollock KH, Simons TR (2010). Experimental investigation of observation error in anuran call surveys.. Journal of Wildlife Management.

[18] Sewell D, Beebee TJC, Griffiths RA (2010). Optimising biodiversity assessments by volunteers: the application of occupancy modelling to large-scale amphibian surveys.. Biological Conservation.

[19] Schmidt BR (2004). Declining amphibian populations: The pitfalls of count data in the study of diversity, distributions, dynamics, and demography.. Herpetological Journal.

[20] Kéry M (2002). Inferring the absence of a species – A case study of snakes.. Journal of Wildlife Management.

[21] Dorazio RM (2007). On the choice of statistical models for estimating occurrence and extinction from animal surveys.. Ecology.

[22] Royle JA, Nichols JD (2003). Estimating abundance from repeated presence-absence data or point counts.. Ecology.

[23] Peterson JT, Bayley PB, Thompson WL (2004). A Bayesian approach to estimating presence when a species is undetected.. Sampling Rare or Elusive Species.

[24] Meier C, Schelbert B (1999). Amphibienschutzkonzept Kanton Aargau.. Aargauer Naturforschende Gesellschaft Mitteilungen.

[25] Schmidt BR (2005). Monitoring the distribution of pond-breeding amphibians when species are detected imperfectly.. Aquatic Conservation.

[26] Vences M (2007). The Amphibian Tree of Life: Ideologie, Chaos or biologische Realität?. Zeitschrift für Feldherpetologie.

[27] MacKenzie DI, Nichols JD, Lachman GB, Droege S, Royle JA (2002). Estimating site occupancy rates when detection probability rates are less than one.. Ecology.

[28] Burnham KP, Anderson DR (2002). Model selection and multimodel inference: a practical information-theoretic approach.

[29] Meyer A, Zumbach S, Schmidt B, Monney J-C (2009). Auf Schlangenspuren und Krötenpfaden: Amphibien und Reptilien der Schweiz.

[30] Sandland RL, Kirkwood GP (1981). Estimation of survival in marked populations with possibly dependent sighting probabilities.. Biometrika.

[31] Fletcher DJ, Fletcher DJ, Manly BFJ (1994). A mark-recapture model in which sighting probability depends on the number of sightings on the previous occasion.. Statistics in ecology and environmental monitoring.

[32] Meyer AH, Schmidt BR, Grossenbacher K (1998). Analysis of three amphibian populations with quarter-century long time-series.. Proceedings of the Royal Society of London, Series B.

[33] Marsh DM (2001). Fluctuations in amphibian populations: a meta-analysis.. Biological Conservation.

[34] Pellet J, Schmidt BR, Fivaz F, Perrin N, Grossenbacher K (2006). Density, climate and varying return points: an analysis of long-term population fluctuations in the threatened European tree frog.. Oecologia.

[35] Salvidio S (2009). Detecting amphibian population cycles: the importance of appropriate statistical analyses.. Biological Conservation.

[36] Schmidt BR, Pellet J (2005). Relative importance of population processes and habitat characteristics in determining site occupancy of two anurans.. Journal of Wildlife Management.

[37] Blankenhorn H-J (1972). Meteorological variables affecting onset and duration of calling in *Hyla arborea* and *Bufo calamita* Laur.. Oecologia.

[38] Pollock KH, Marsh H, Bailey LL, Farnsworth GL, Simons TR, Thompson WL (2004). Separating components of detection probability in abundance estimation: An overview with diverse examples.. Sampling Rare or Elusive Species.

[39] Nichols JD, Thomas L, Conn PB, Thompson DL, Cooch EG, Conroy MJ (2008). Inferences about landbird abundance from count data: recent advances and future directions.. Modeling demographic processes in marked populations.

[40] Royle JA (2004). *N*-mixture models for estimating population size from spatially replicated counts.. Biometrics.

[41] Royle JA, Dorazio RM (2006). Hierarchical models of animal abundance and occurrence.. Journal of Agricultural, Biological and Environmental Statistics.

[42] Kéry M, Royle JA, Schmid H (2005). Modeling avian abundance from replicated counts using binomial mixture models.. Ecological Applications.

[43] Kéry M, Dorazio RM, Soldaat L, van Strien A, Zuiderwijk A (2009). Trend estimation in populations with imperfect detection.. Journal of Applied Ecology.

[44] Pellet J (2007). Seasonal variation in detectability of butterflies surveyed with Pollard walks.. Journal of Insect Conservation.

[45] Royle JA (2006). Site occupancy models with heterogeneous detection probabilities.. Biometrics.

[46] Alpizar-Jara R, Nichols JD, Hines JE, Sauer JR, Pollock KH (2004). The relationship between species detection probability and local extinction probability.. Oecologia.

