# Supplementary material for: Population Size Influences Amphibian Detection Probability: Implications for Biodiversity Monitoring Programs
Source: PLoS One. 2011 Dec 2;6(12):e28244. doi: 10.1371/journal.pone.0028244 (PMC3229540; doi:10.1371/journal.pone.0028244)
Supplement: Table S1 — Models sets and model selection results for each species. The table lists all candidate models for all species and shows the results of the model selection process (ΔAICc, Akaike weights, number of parameters (K) and -2log-likelihood). (PDF) [file pone.0028244.s001.pdf]

**Table S1** Models sets and model selection results for each species.

| **Model** | **ΔAICc [a]** | **Akaike weight [b]** | **K [c]** | **2log-likelihood** |
|---|---|---|---|---|
| *P. esculentus* | | | | |
| psi(.), p(PASTPOP, TIMESINCE) | 0.00 | 0.579 | 5 | 424.85 |
| psi(.), p(PASTPOP) | 0.64 | 0.420 | 4 | 427.49 |
| psi(.), p(AIRT) | 16.18 | 0.000 | 4 | 443.03 |
| psi(.), p(VISIT, VISITSQ) | 17.26 | 0.000 | 5 | 442.11 |
| psi(.), p(SOILT) | 18.47 | 0.000 | 4 | 445.31 |
| psi(.), p(WIND) | 19.01 | 0.000 | 4 | 445.85 |
| psi(.), p(.) | 21.90 | 0.000 | 2 | 452.74 |
| psi(.), p(REED) | 22.72 | 0.000 | 4 | 449.56 |
| psi(.), p(WSURFACE) | 22.77 | 0.000 | 4 | 449.61 |
| psi(.), p(FLOATINGP) | 23.66 | 0.000 | 4 | 450.50 |
| psi(.), p(RESTRICTED) | 24.31 | 0.000 | 4 | 451.15 |
| psi(.), p(RAIN) | 24.34 | 0.000 | 4 | 451.19 |
| psi(.), p(UNDERWP) | 24.36 | 0.000 | 4 | 451.21 |
| *A. obstetricans* | | | | |
| psi(.), p(SOILT) | 0.00 | 0.347 | 4 | 279.78 |
| psi(.), p(AIRT) | 0.18 | 0.317 | 4 | 279.95 |
| psi(.), p(PASTPOP) | 2.35 | 0.107 | 4 | 282.12 |
| psi(.), p(VISIT, VISITSQ) | 2.99 | 0.077 | 5 | 280.76 |
| psi(.), p(PASTPOP, TIMESINCE) | 4.34 | 0.039 | 5 | 282.11 |
| psi(.), p(WSURFACE) | 4.46 | 0.037 | 4 | 284.24 |
| psi(.), p(RESTRICTED) | 4.81 | 0.031 | 4 | 284.59 |

| | | | | |
|---|---|---|---|---|
| psi(.), p(WIND) | 4.99 | 0.028 | 4 | 284.77 |
| psi(.), p(RAIN) | 6.48 | 0.013 | 4 | 286.25 |
| psi(.), p(.) | 27.90 | 0.000 | 2 | 311.68 |
| *B. calamita* | | | | |
| psi(.), p(PASTPOP, TIMESINCE) | 0.00 | 0.255 | 5 | 167.59 |
| psi(.), p(PASTPOP) | 1.00 | 0.154 | 4 | 170.58 |
| psi(.), p(SOILT) | 1.48 | 0.121 | 4 | 171.07 |
| psi(.), p(AIRT) | 1.62 | 0.113 | 4 | 171.20 |
| psi(.), p(RAIN) | 1.97 | 0.095 | 4 | 171.56 |
| psi(.), p(VISIT, VISITSQ) | 1.98 | 0.094 | 5 | 169.56 |
| psi(.), p(WSURFACE) | 2.88 | 0.060 | 4 | 172.47 |
| psi(.), p(WIND) | 3.16 | 0.052 | 4 | 172.74 |
| psi(.), p(RESTRICTED) | 3.24 | 0.052 | 4 | 172.82 |
| psi(.), p(.) | 10.04 | 0.001 | 2 | 183.62 |
| *B. variegata* | | | | |
| psi(.), p(WIND) | 0.00 | 0.518 | 4 | 289.22 |
| psi(.), p(AIRT) | 0.57 | 0.389 | 4 | 298.80 |
| psi(.), p(VISIT, VISITSQ) | 5.70 | 0.030 | 5 | 301.92 |
| psi(.), p(SOILT) | 6.15 | 0.023 | 4 | 304.37 |
| psi(.), p(RAIN) | 7.60 | 0.011 | 4 | 305.83 |
| psi(.), p(WSURFACE) | 8.10 | 0.009 | 4 | 306.33 |
| psi(.), p(PASTPOP) | 8.58 | 0.007 | 4 | 306.81 |
| psi(.), p(RESTRICTED) | 8.61 | 0.007 | 4 | 306.83 |
| psi(.), p(PASTPOP, TIMESINCE) | 9.84 | 0.003 | 5 | 306.07 |
| psi(.), p(.) | 24.70 | 0.000 | 2 | 326.93 |

| | | | | |
|---|---|---|---|---|
| *M. alpestris* | | | | |
| psi(.), p(PASTPOP) | 0.00 | 0.236 | 4 | 513.53 |
| psi(.), p(VISIT, VISITSQ) | 1.03 | 0.141 | 5 | 512.55 |
| psi(.), p(SOILT) | 1.06 | 0.139 | 4 | 514.59 |
| psi(.), p(PASTPOP, TIMESINCE) | 1.15 | 0.133 | 5 | 512.67 |
| psi(.), p(RAIN) | 1.66 | 0.103 | 4 | 515.19 |
| psi(.), p(AIRT) | 2.00 | 0.087 | 4 | 515.52 |
| psi(.), p(FLOATINGP) | 3.01 | 0.052 | 4 | 516.53 |
| psi(.), p(UNDERWP) | 3.90 | 0.033 | 4 | 517.43 |
| psi(.), p(WSURFACE) | 4.38 | 0.026 | 4 | 517.91 |
| psi(.), p(REED) | 4.62 | 0.023 | 4 | 518.15 |
| psi(.), p(RESTRICTED) | 4.71 | 0.022 | 4 | 518.24 |
| psi(.), p(.) | 83.36 | 0.000 | 2 | 600.89 |
| *T. cristatus* | | | | |
| psi(.), p(PASTPOP, TIMESINCE) | 0.00 | 0.738 | 5 | 112.12 |
| psi(.), p(PASTPOP) | 3.17 | 0.151 | 4 | 117.29 |
| psi(.), p(FLOATINGP) | 5.53 | 0.046 | 4 | 119.66 |
| psi(.), p(RESTRICTED) | 6.65 | 0.026 | 4 | 120.77 |
| psi(.), p(UNDERWP) | 6.72 | 0.025 | 4 | 120.84 |
| psi(.), p(AIRT) | 11.05 | 0.002 | 4 | 125.18 |
| psi(.), p(SOILT) | 11.48 | 0.002 | 4 | 125.61 |
| psi(.), p(RAIN) | 11.80 | 0.002 | 4 | 125.92 |
| psi(.), p(REED) | 12.42 | 0.001 | 4 | 126.55 |
| psi(.), p(WSURFACE) | 12.74 | 0.001 | 4 | 126.87 |
| psi(.), p(VISIT, VISITSQ) | 12.81 | 0.001 | 5 | 124.94 |

| | | | | |
|---|---|---|---|---|
| psi(.), p(.) | 21.65 | 0.000 | 2 | 139.78 |

[a] ΔAICc is the difference between the AICc of the best model and the focal model.

[b] K is the number of parameters included in the model.

[c] The sum of all Akaike weights in a set of candidate models is 1. The higher the weight, the better the model is supported by the data.
